# Systematic review on the application of wearable inertial sensors to quantify everyday life motor activity in people with mobility impairments

**DOI:** 10.1186/s12984-020-00779-y

**Published:** 2020-11-04

**Authors:** Fabian Marcel Rast, Rob Labruyère

**Affiliations:** 1 Swiss Children’s Rehab, University Children’s Hospital Zurich, Mühlebergstrasse 104, 8910 Affoltern am Albis, Switzerland; 2 Children’s Research Center, University Children’s Hospital of Zurich, University of Zurich, Zurich, Switzerland; 3 Rehabilitation Engineering Laboratory, Department of Health Sciences and Technology, ETH Zurich, Zurich, Switzerland

**Keywords:** Disabled persons, Patients, Rehabilitation, Accelerometer, Gyroscope, Inertial measurement unit, Algorithms, Pattern recognition, Machine learning, Activities of daily living

## Abstract

**Background:**

Recent advances in wearable sensor technologies enable objective and long-term monitoring of motor activities in a patient’s habitual environment. People with mobility impairments require appropriate data processing algorithms that deal with their altered movement patterns and determine clinically meaningful outcome measures. Over the years, a large variety of algorithms have been published and this review provides an overview of their outcome measures, the concepts of the algorithms, the type and placement of required sensors as well as the investigated patient populations and measurement properties.

**Methods:**

A systematic search was conducted in MEDLINE, EMBASE, and SCOPUS in October 2019. The search strategy was designed to identify studies that (1) involved people with mobility impairments, (2) used wearable inertial sensors, (3) provided a description of the underlying algorithm, and (4) quantified an aspect of everyday life motor activity. The two review authors independently screened the search hits for eligibility and conducted the data extraction for the narrative review.

**Results:**

Ninety-five studies were included in this review. They covered a large variety of outcome measures and algorithms which can be grouped into four categories: (1) maintaining and changing a body position, (2) walking and moving, (3) moving around using a wheelchair, and (4) activities that involve the upper extremity. The validity or reproducibility of these outcomes measures was investigated in fourteen different patient populations. Most of the studies evaluated the algorithm’s accuracy to detect certain activities in unlabeled raw data. The type and placement of required sensor technologies depends on the activity and outcome measure and are thoroughly described in this review. The usability of the applied sensor setups was rarely reported.

**Conclusion:**

This systematic review provides a comprehensive overview of applications of wearable inertial sensors to quantify everyday life motor activity in people with mobility impairments. It summarizes the state-of-the-art, it provides quick access to the relevant literature, and it enables the identification of gaps for the evaluation of existing and the development of new algorithms.

## Background

The protocol of this systematic review was published in advance [1], and the following introduction is an adapted and extended version of the introduction of that protocol.

People with mobility impairments may have difficulties in executing activities of daily living (activity limitations), or they may experience problems in involvement in life situations (participation restrictions) [2]. Rehabilitation services aim to improve these people’s abilities or make changes to their environment [3], to achieve a high level of independence and eventually increase the quality of life. Clinical assessments to estimate patients’ abilities and their rehabilitation progress are generally conducted in a standardized environment at a single time. Thus, they do not incorporate environmental and cognitive challenges of a patient’s habitual environment [4] and might be inaccurate when the symptoms of the patient fluctuate over time [5].

Recent advances in wearable sensor technologies enable objective and long-term monitoring of motor activities in a patient’s habitual environment. They provide an opportunity to overcome the aforementioned limitations of clinical assessments and complement their outcome measures. Accelerometers are the most commonly used wearable devices to quantify everyday life motor activity in clinical trials and clinical practice [6, 7]. Conventional outcome measures of accelerometers are activity counts as well as intensity levels and energy expenditure estimations based on cut-points of these counts [8]. These measures provide relevant information about whole-body physical activity, but they are non-specific and cannot determine movement patterns and types of activities performed [9]. In contrast, using a combination of several inertial sensors, such as accelerometers and gyroscopes, together with sophisticated data processing algorithms, allows estimating the quantity and other characteristics of everyday life motor activities [10]. Additional sensor technology such as magnetometers, barometers, wearable cameras, and heart rate monitors measure environmental factors or physiological responses to motor activities and can be combined with inertial sensors to gain further details about patients’ activities [11, 12]. Technological progress in the field of micro-electromechanical systems has made these devices small-sized, cost-effective, energy-efficient, and thus applicable for continuous long-term monitoring in unsupervised conditions [10]. However, continuous long-term monitoring generates a tremendous amount of unlabeled data that requires appropriate data processing algorithms to determine clinically meaningful outcome measures of everyday life motor activity. Typically, these algorithms detect a certain activity in unlabeled data as a first step (e.g., walking bouts or grasping an object) and then determine a measure to quantify the previously detected activity as a second step (e.g., walking speed or number of grasping activities).

The relevance of these outcome measures depends on end-users’ perspectives and may be different for people with mobility impairments compared to non-disabled individuals. For example, the amount of limping, use of assistive devices, and daily activity of affected limbs are more relevant to the former population. Altered movement patterns can also be a challenge for data processing algorithms [13, 14] and thus the transferability of algorithms which were evaluated in non-disabled individuals to people with mobility impairments could be limited. Therefore, this review focused on the application of inertial sensor technologies to quantify everyday life motor activity in people with mobility impairments and provides an overview of existing outcome measures as well as their underlying data processing algorithms. Specifically, the following research questions were addressed: (1) Which outcome measures have been used to quantify everyday life motor activity of people with mobility impairments under free-living conditions, and what are their corresponding data processing algorithms? (2) Which inertial sensor technology (accelerometer or gyroscope), possibly in combination with additional wearable sensor technology, is required to assess these measures? (3) Where need inertial sensors be placed to assess these measures and minimally restrict activities of daily living? (4) In which patient populations were these measures applied, and were they and the required sensor system evaluated in terms of validity, reproducibility, or usability?

## Methods

The detailed protocol of this review was published in advance [1] and its method section is roughly summarized in the following paragraphs.

The systematic search was conducted in three databases: MEDLINE, EMBASE, and SCOPUS. The selected search terms can be grouped into five categories: (1) study population (e.g., “patient”, “stroke”, etc.), (2) measurement tool (e.g., “accelerometer”, “gyroscope”, etc.), (3) data processing algorithm (e.g., “algorithm”, “signal processing”, etc.), (4) free-living condition (e.g., “everyday life”, “daily living”, etc.), and (5) two terms which incorporate categories three and four (“activity classification” and “activity recognition”). A first search was conducted in July 2017 and repeated in October 2019.

Title and abstracts (first step), as well as full-text articles (second step) were screened by the two review authors independently to identify articles that met the following eligibility criteria: (1) The study population involved children, adolescents, or adults with a diagnosed orthopedic or neurological mobility impairment or people who need assistive devices in their daily life activities, (2) the article used a measurement tool that incorporates a wearable accelerometer, gyroscope, or both, i.e., inertial measurement unit (IMU), and optionally includes additional sensors, (3) the article described the underlying data processing algorithm reproducibly or cited a publicly available reference, and (4) the output of the algorithm is a measure that quantifies an aspect of everyday life motor activity. Whole-body activity counts, as well as physical activity levels and energy expenditure based on thresholds of these counts, were not considered for this review, as they have already been well investigated [15, 16].

The used outcome measures and the method of the underlying data processing algorithm, the type and placement of required sensor technology, the study population as well as the study design were extracted from all included articles. Some studies investigated more than one sensor setup and data processing algorithm. In that case, only the method with the best performance or the recommended method was included in this review. If the outcome measures were not explicitly mentioned or described in the article, which was often the case in activity classification studies, it was assumed that activity detection enables to determine the duration of the activity or count the number of repetitions. The measures were then retrospectively grouped into four categories: (1) Maintaining and changing a body position, (2) walking and moving, (3) moving around using a wheelchair, and (4) activities that involve the upper extremity. The sensor placements were simplified by assigning the exact positions to one of the following body segments: head, trunk, upper arm, forearm, hand, pelvis, thigh, shank, foot, and assistive devices. Thus, sensors that were placed above the lateral malleoli and on the fifth lumbar vertebra were assigned to the shank and pelvis segment, respectively. To address the second part of the fourth research question, the study designs were allocated to one or several of seven different categories: *Classification accuracy studies* investigated the performance of the algorithm to recognize activities, while *technical validity studies* determined the accuracy of activity-related measures, both with regard to a reference method. *Clinical validity studies* correlated the outcome of the sensor system with the outcome of a clinical assessment. *Between-day reliability studies* investigated the consistency of the outcome when measuring it on two different days. *Case/control studies* compared the outcome between the target population and a control group. *Interventional studies* used the outcome to evaluate the effectiveness of an intervention, and *observational studies* incorporated different designs such as analyzing the changes of the outcome over time or comparing several outcomes within the same subject. Besides, it was determined if the studies assessed the usability of the sensor systems.

## Results

### Overview

**Fig. 1:**
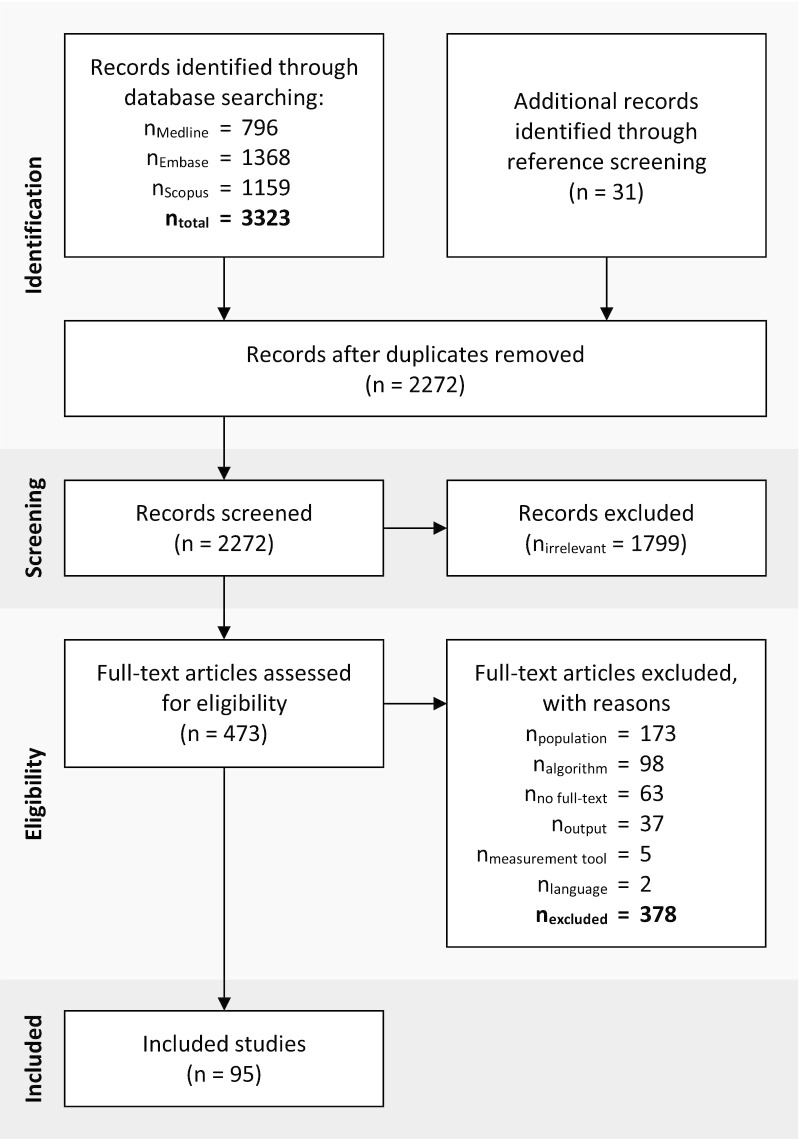
Flow diagram according to the Preferred Reporting Items for Systematic Reviews and Meta-Analyses (PRISMA) [120]

The systematic search revealed 2272 hits, of which 31 were added retrospectively through reference screening of the included articles. After title and abstract screening, 473 articles remained for full-text screening, and, eventually, 95 articles fulfilled the predetermined eligibility criteria. The complete flow diagram of the screening procedure is shown in Fig. 1. The main reason for exclusion was the study population, with 46% of all excluded articles. Many research projects developed a new algorithm to monitor motor activities in daily life and conducted a preliminary study with healthy subjects. These studies were not considered in this review, except for one study that recruited able-bodied individuals which performed an activity circuit in a wheelchair [17]. The second most frequent exclusion criterion was the algorithm with 26%. It was either not described reproducibly (e.g., in cases of proprietary algorithms of commercial parties) or not applicable to unlabeled data.

**Fig. 2:**
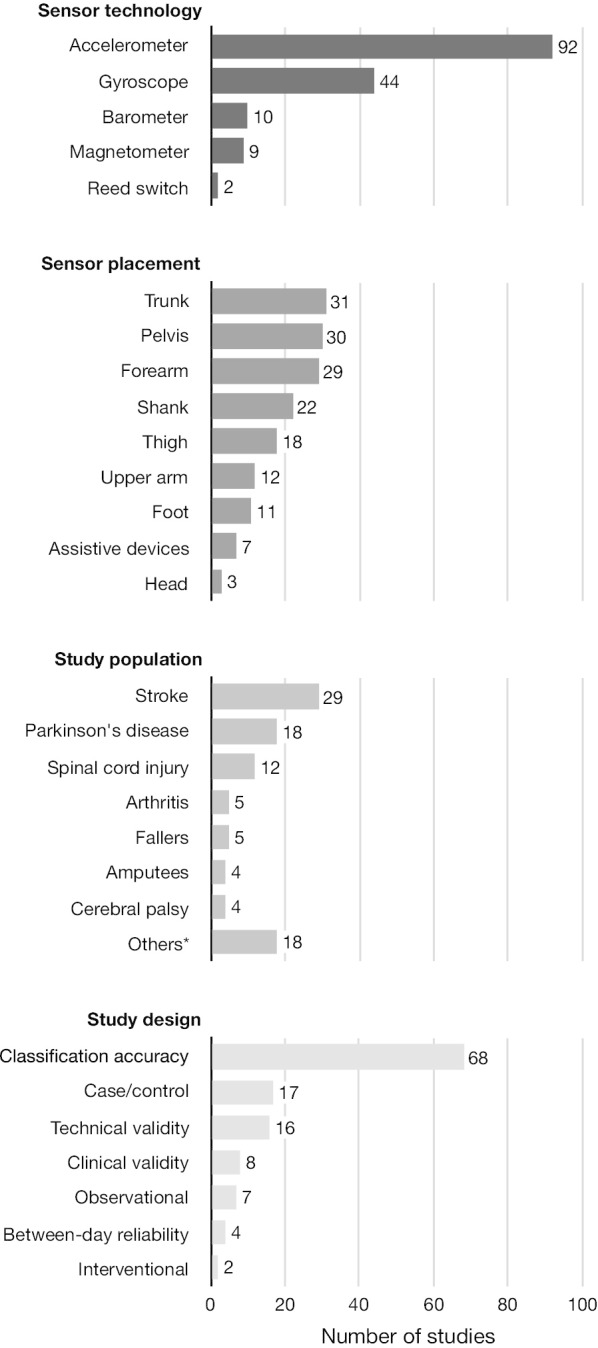
Frequency distribution of the used sensor technologies, of the body segments on which sensors were placed, of the study population in which the sensors were applied, and of the used study designs to evaluate the outcome measures

An overview of the used sensor technologies, the body segments on which sensors were placed, the study population in which the sensors were applied, and the used study designs for evaluating the outcome measures is provided in Fig. 2. Note that most of the studies were allocated to several of the chosen categories.

#### Sensor technologies

All studies used an accelerometer, a gyroscope, or both (inclusion criteria) with a clear preference for accelerometers. These sensor technologies were combined with a barometric pressure sensor to detect changes in altitude, a magnetometer to measure the orientation relative to the earth’s magnetic field, and a reed switch on the spokes of the wheelchair to determine revolutions of the wheel. Six studies used an instrumented insole with force-sensitive sensors [18–23], and two studies used a first-person camera [24, 25], all in combination with inertial sensors. These eight studies were not further considered in this review since they did not use inertial sensors as their primary measurement tool.

#### Sensor placement

The sensors were most frequently placed on the trunk, the pelvis, and the forearm but also on other body segments and on assistive devices. The frequency of chosen sensor positions depended mainly on the outcome measures. Studies that used outcomes related to body positions preferred a sensor on the trunk or a combination of trunk and thigh sensors. In contrast, studies that used outcomes related to activities of the upper extremities (incl. wheeling) placed the sensors on the arms with a clear preference of wrist sensors. There was no clear preference for sensor placement in studies with gait-related outcomes. Sensors were placed on the trunk, the pelvis, the shanks, and the feet. The sensor placement, in general, is strongly related to the underlying algorithm and, therefore, more thoroughly described in the subsequent chapters.

#### Study populations

Wearable inertial sensors were most frequently applied in stroke survivors, in patients with Parkinson’s disease, and in patients with spinal cord injury. Fourteen different study populations were identified, which highlights the wide range of applications of wearable inertial sensors to quantify everyday life motor activity in people with mobility impairments.

#### Study designs

In terms of validity, the majority of the included studies evaluated the algorithm’s activity classification accuracy. The methods of these studies differed considerably. Measurements were conducted under laboratory or free-living conditions. The number of sensors ranged from 1 to 17 and the number of classes/activities from 1 to 11. Moreover, the methods to split the data into training and testing samples varied, and the studies used inconsistent metrics to report their results. Technical and clinical validity studies were conducted less frequently. Technical validity studies determined predominantly the accuracy of gait parameters. Sensor-based outcome measures were compared to those of pressure-sensitive walkways, video recordings, stopwatches, or other validated sensor systems. In contrast, the clinical validity studies compared their sensor-based outcome measures to those of clinical assessments. These comparisons were unique for each clinical validity study of this review. Clinical studies were less frequent than validity studies. Here, sensor-based outcome measures were often applied in case/control studies, followed by observational and interventional studies. In terms of reproducibility, four studies determined the between-day reliability of their outcome measure. All of them evaluated gait-related outcomes, but they differed considerably in the chosen setting. Two studies assessed the usability of a sensor system by reporting inconvenience [26] and adverse events [27], respectively, while eight studies reported the wearing time of the sensors in daily life [28–35].

#### Outcome measures and underlying algorithms

All outcomes, as well as the underlying type and placement of sensors, are thoroughly described in the subsequent chapters. Each chapter is complemented with a table that provides a list of all outcome measures and how they were investigated in terms of study populations and study designs (Tables 1, 2, 3, 4). The underlying data processing algorithms to detect activities in unlabeled data of this review followed either a biomechanical or a statistical machine learning approach. The former approach uses explicit, and a priori defined features that are specific to certain activities (e.g., the orientation of the thigh during sitting). The concepts of this approach are described in the following chapters. The latter approach uses many unspecific features in combination with standard machine learning algorithms. A description of these algorithms is provided elsewhere [36], and the detected activity classes, as well as the used sensor type and placement, are listed in Table 5.

**Table 1:** Overview of activities and measures regarding maintaining and changing a body position as well as the corresponding study populations and study designs

Activity	Measure	Diagnosis/impairment group	Study design
Maintaining a body position			
Lying	Duration	Amputees [37, 38], arthritis [48, 60], cerebral palsy [33, 61], Duchenne muscular atrophy [26], frail people [35], multiple sclerosis [39], Parkinson's disease [40–42, 49], spinal cord injury [43, 50, 62], stroke [44–47]	Between-day reliability [33], case/control [35, 49], classification accuracy [26, 37–41, 43–48, 50, 60–62], interventional [26]
	Knee angle	Multiple sclerosis [39]	Technical validity [39]
Sitting	Duration	Amputees [37, 38, 51], arthritis [31, 48, 60], cerebral palsy [33, 61], Duchenne muscular atrophy [26], frail people [35], multiple sclerosis [39], Parkinson's disease [40–42, 49, 52, 53], post-surgery [55], rotator cuff syndrome [30, 56], spinal cord injury [43, 50, 62], stroke [44–47, 57–59], miscellaneous [54]	Between-day reliability [33], case/control [30, 31, 35, 49, 56], classification accuracy [26, 37–48, 50–55, 57–62], interventional [26], observational [58]
	Knee angle	Multiple sclerosis [39]	Technical validity [39]
Standing	Duration	Amputees [37, 38], arthritis [31, 48, 60], cerebral palsy [33, 61], Duchenne muscular atrophy [26], frail people [35], multiple sclerosis [39], Parkinson's disease [40–42, 49, 52, 53], post-surgery [55], rotator cuff syndrome [30, 56], spinal cord injury [43, 50], stroke [44–47, 58, 59], miscellaneous [54]	Between-day reliability [33], case/control [30, 31, 35, 49, 56], classification accuracy [26, 37–48, 50, 52–55, 58–61], interventional [26], observational [58]
	Knee angle	Multiple sclerosis [39]	Technical validity [39]
Changing a body position			
Lying ⇔ sitting	# of transitions	Arthritis [48], chronic pain [70], stroke [57]	Classification accuracy [48, 57, 70]
Lying ⇔ standing	# of transitions	Arthritis [48]	Classification accuracy [48]
Sitting ⇔ standing/walking	# of transitions	Arthritis [48], chronic pain [70], fallers [71], frail people [64], Parkinson’s disease [40, 41, 53, 65, 67, 68, 72, 73], post-surgery [66], risk of falling [63], stroke [57, 59, 69]	Case/control [64, 71, 73], classification accuracy [40, 41, 48, 53, 57, 59, 63, 65–70, 72, 73], clinical validity [40, 63]
	Duration	Fallers [71, 74], frail people [64], Parkinson’s disease [40, 68, 73, 75], risk of falling [63]	Case/control [64, 71, 73–75], clinical validity [40, 63, 75], technical validity [63, 68]
	Trunk tilt angle	Fallers [71], frail people [64]	Case/control [64, 71]
	Smoothness	Fallers [74], Parkinson’s disease [75]	Case/control [74, 75], clinical validity [75]
	Others^1^	Fallers [74], frail people [64], Parkinson’s disease [40, 75]	Case/control [64, 74, 75], clinical validity [40, 75]
Transferring oneself			
Transferring oneself while sitting	Duration	Spinal cord injury [62]	Classification accuracy [62]

^1^Range and maxima of acceleration and gyroscope signals of the trunk and the pelvis

**Table 2:** Overview of activities and measures regarding walking and moving as well as the corresponding study populations and study designs

Activity	Measure	Diagnosis / Impairment group	Study design
Walking			
Walking	Duration	Amputees [37, 38, 51], arthritis [31, 48, 60, 87], cerebral palsy [33, 61, 92], chronic pain [70], Duchenne muscular atrophy [26], fallers [28], frail people [35], multiple sclerosis [39], Parkinson’s disease [40–42, 49, 52, 53, 68, 72, 79], post-surgery [55], risk of falling [63], spinal cord injury [43, 50, 90], stroke [27, 44–47, 57, 58, 83–85, 88, 91], miscellaneous [54, 78]	Between-day reliability [27, 28, 33], case/control [28, 31, 35, 49], classification accuracy [26, 37–48, 50–55, 57, 58, 60, 61, 68, 70, 72, 78, 79, 83–85, 87, 88, 90–92], interventional [26], observational [58, 80], technical validity [63, 68]
	# of steps / cadence	Arthritis [31, 60], cerebral palsy [33, 92], chronic pain [93], Duchenne muscular atrophy [26], fallers [28], frail people [35], multiple sclerosis [39], Parkinson’s disease [77, 80, 86], risk of falling [63], stroke [27]	Between-day reliability [27, 28, 33, 93], case/control [28, 31, 35, 86, 93], clinical validity [93], interventional [26], observational [80], technical validity [26, 27, 39, 60, 63, 77, 92]
	Temporal gait parameters	Cerebral palsy [33], Parkinson’s disease [86], stroke [27]	Between-day reliability [27, 33], case/control [86], technical validity [27]
	Walking speed / stride length	Fallers [28], cerebral palsy [33, 61], chronic pain [70, 93], Parkinson’s disease [40, 86], stroke [27, 58, 91]	Between-day reliability [27, 28, 33, 93], case/control [28, 86, 93], classification accuracy [61], clinical validity [40, 93], observational [58], technical validity [27, 70, 91]
	Walking stability	Chronic pain [93], fallers [81, 82]	Between-day reliability [93], case/control [81, 82, 93], clinical validity [93]
	Foot clearance	Stroke [58]	Observational [58]
	Knee angle	Cerebral palsy [33], multiple sclerosis [39]	Between-day reliability [33], technical validity [39]
Walking sideways	Duration	Stroke [59]	Classification accuracy [59]
Walking while carrying an object	Duration	Arthritis [87], stroke [88]	Classification accuracy [87, 88]
Turning	# of turns	Parkinson’s disease [49, 68, 72, 79, 94, 95]	Case/control [49, 79, 94], classification accuracy [68, 72, 95], clinical validity [94]
	Duration	Arthritis [87], Parkinson’s disease [68, 72, 79, 94, 95], stroke [88]	Case/control [79, 94], classification accuracy [79, 87, 88], clinical validity [94], technical validity [68, 72, 95]
	Turning angle	Parkinson’s disease [79, 94, 95]	Case/control [79, 94], clinical validity [94], technical validity [95]
	Turning speed	Parkinson’s disease [79, 94]	Case/control [79, 94], clinical validity [94]
	Others^1^	Parkinson’s disease [79, 94]	Case/control [79, 94], clinical validity [94]
Walking on sloping surfaces	Duration	Arthritis [87], stroke [47, 88]	Classification accuracy [47, 87, 88]
Stair climbing			
Stair climbing	Duration	Amputees [51], arthritis [60, 87], post-surgery [55], spinal cord injury [43, 50], stroke [44–47, 59, 83, 84, 88], miscellaneous [54, 96]	Classification accuracy [43–47, 50, 51, 54, 55, 59, 60, 83, 84, 87, 88, 96]
	# of steps	Arthritis [60]	Technical validity [60]
	Step-by-step vs. step-over-step	Stroke [83]	Classification accuracy [83]
Running			
Running	Duration	Amputees [51]	Classification accuracy [51]

^1^Smoothness, mediolateral range of trunk acceleration, and number of steps to complete a turn

**Table 3:** Overview of activities and measures regarding wheeling as well as the corresponding study populations and study designs

Activity	Measure	Diagnosis/impairment group	Study design
Moving around using a wheelchair			
Self-propelled wheeling	Duration	Able-bodied wheelchair users [17], spinal cord injury [32, 62, 97–101]	Classification accuracy [17, 32, 62, 97–101]
	Distance	Spinal cord injury [29, 32]	Clinical validity [29], technical validity [32]
	Speed	Able-bodied wheelchair users [17], spinal cord injury [29]	Classification accuracy [17], clinical validity [29]
	# of strokes/stroke frequency	Spinal cord injury [102]	Interventional [102], technical validity [102]
Maneuvering	Duration	Able-bodied wheelchair users [17], spinal cord injury [32]	Classification accuracy [17, 32]
Playing basketball	Duration	Spinal cord injury [101]	Classification accuracy [101]

**Table 4:** Overview of activities and measures regarding upper extremities as well as the corresponding study populations and study designs

Activity	Measure	Diagnosis/impairment group	Study design
Non-specific hand and arm use			
n/a	Duration/laterality	Parkinson’s disease [40], Rotator cuff syndrome [30], stroke [46, 47, 58, 89, 103, 104]	Case/control [103], classification accuracy [40, 46, 47, 58, 103, 104], clinical validity [40, 89, 103], observational [30]
n/a	Entropy	Arthritis [105]	Case/control [105]
n/a	Range of motion		
	Shoulder	Rotator cuff syndrome [56], stroke [34, 106]	Observational [34, 56, 106]
	Elbow	Stroke [34]	Observational [34]
	Wrist and finger	Stroke [107]	Observational [107]
Specific hand and arm movements			
Reaching	# and duration of reaching activities	Parkinson’s disease [72], stroke [34, 108–111]	Classification accuracy [72, 108–111], observational [34]
	Reaching distance	Stroke [58]	Observational [58]
	Reaching direction	Parkinson’s disease [72], stroke [58]	Classification accuracy [72], observational [58]
Lifting sth. to the mouth	Duration	Stroke [57, 108–111]	Classification accuracy [57, 108–111]
Pouring sth. (pro-/supination)	Duration	Stroke [108–111]	Classification accuracy [108–111]
Specific hand and arm activities			
Writing and reading	Duration	Parkinson’s disease [42]	Classification accuracy [42]
Opening a door	Duration	Arthritis [87], stroke [88]	Classification accuracy [87, 88]
Hair combing	Duration	Stroke [57, 112]	Classification accuracy [57, 112]
Eating	Duration	Parkinson’s disease [42], stroke [112], miscellaneous [113]	Classification accuracy [42, 112, 113]
Drinking	Duration	Stroke [112]	Classification accuracy [112]
Tooth brushing, shirt buttoning, pant lifting, food cutting	Duration	Stroke [57]	Classification accuracy [57]

**Table 5:** Activity classes and sensor placement of the statistical machine learning approaches

First author	^#^ of classes	Names of classes	^#^ of sensors	Sensor positions	Type of sensors
Ahmadi [61]	4	Sedentary (lying and sitting), standing, comfortable walking, and brisk walking	2	Pelvis and forearm (dominant side)	ACC
Albert [52]	4	Lying, sitting, standing, and walking	1	Pelvis	ACC
Albert [43], Sok [50]	6	Lying, sitting, standing, walking, stair climbing, and wheeling	1	Pelvis	ACC
Andreu-Perez [48]	10	Lying, sitting, standing, lying-to-sitting, sitting-to-lying, lying-standing, standing-to-lying, sitting-to-standing, standing-to-sitting, and walking	1	Pelvis	ACC
Biswas [110, 111]	3	Reaching and retrieving, lifting cup to mouth, and pouring and (un)locking	1	Forearm (affected side)	ACC and GYRO
Brogioli [29], Popp [32]	2	Passive and active wheeling	4	Trunk, forearm (bilateral), and wheelchair	ACC and GYRO
Capela [46]	6	Lying, sitting, standing, walking, stair climbing, and activities of daily living	1	Pelvis	ACC
Capela [47]	6	Lying, sitting, standing, walking, stair climbing, and activities of daily living	1	Pelvis	ACC and GYRO
Cheng [49]	6	Lying, sitting, standing, walking, stair climbing, and running	1	Pelvis	ACC and GYRO
Ding [100]	4	Static, non-wheeling activity, passive wheeling, and active wheeling	2	Forearm (dominant side) and wheelchair	ACC (forearm) and RS (wheelchair)
Fortune [99]	3	Static, non-wheeling activity, and active wheeling	3	Trunk and upper arm (bilateral)	ACC and GYRO
García-Massó [62]	5	Sedentary (lying, sitting, and passive wheeling), transferring while sitting, active wheeling, housework, and arm-ergometer	2	Forearm (bilateral)	ACC
Hester [87]	10	Walking, walking on uneven surfaces, walking up a ramp, walking down a ramp, stair climbing up, stair climbing down, walking over an object, turning, walking while carrying an object, and opening a door	5	Forearm (bilateral), shank (bilateral), and walking aid	ACC and GYRO
Hester [88]	10	Walking, walking on uneven surfaces, walking up a ramp, walking down a ramp, stair climbing up, stair climbing down, walking over an object, turning, walking while carrying an object, and opening a door	2	Shank (right) and walking aid	ACC
Hiremath [101]	7	Static, passive wheeling, active wheeling, housework, activities of daily living, arm-ergometer, and playing basketball	3	Upper arm (dominant side), forearm (dominant side), and wheelchair	ACC (upper arm and forearm) and GYRO (wheelchair)
Jalloul [42]	7	Lying, sitting, standing, walking, eating, writing, and reading	6	Neck, upper arm (unilateral), forearm (unilateral), pelvis, thigh (unilateral), and shank (unilateral)	ACC and GYRO and MAG
Kiani [37, 38]	6	Lying, sitting, standing, transitions, walking, and unlabeled	3	Trunk, thigh (left and right)	ACC
Laudanski [83]	5	Walking, stair climbing up (step over step), stair climbing up (step by step), stair climbing down (step over step), and stair climbing down (step by step)	2	Shank (bilateral)	ACC and GYRO
Leuenberger [84]	3	Walking, stair climbing up, and stair climbing down	5	Trunk, forearm (bilateral), and shank (bilateral)	ACC and BARO
Lonini [54]	5	Sitting, standing, walking, stair climbing up, and stair climbing down	1	Pelvis	ACC
O’Brien [45]	6	Lying, sitting, standing, walking, stair climbing up, and stair climbing down	1	Pelvis	ACC and GYRO and BARO
Popp [90]	4	Sedentary, low intensity, high intensity, and walking	3	Trunk, forearm (affected side), and shank (non-affected side)	GYRO and BARO
Popp [98]	3	Low intensity, high intensity, and active wheeling	4	Trunk, forearm (bilateral), and wheelchair	ACC and GYRO
Recher [59]	7	Sitting, standing, sitting-to-standing, standing-to-sitting, walking sideways, stair climbing, and moving objects	8	Trunk, pelvis, thigh (bilateral), shank (bilateral), and foot (bilateral)	ACC and GYRO and MAG
Roy [57]	11	Supine-to-sitting, sitting, sitting-to-standing, walking, tooth brushing, hair combing, bowel movement, shirt buttoning, pant lifting, food cutting, and food lifting	8	Abdomen, lower back (bilateral), upper arm (bilateral), forearm (bilateral), thigh (affected side)	ACC
Seiter [113]	6	Resting (lying & sitting), eating and leisure, cognitive training, medical fitness, kitchen work, and motor training	3	Forearm (bilateral) and thigh (non-affected side)	ACC
Teknomo [51]	4	Sitting, walking, stair climbing, and running	1	Shank (non-affected side)	ACC
Wade [55]	4	Sitting, standing, walking, and stair climbing	5	Pelvis, thigh (bilateral), shank (bilateral)	ACC
Wu [85], Xu [91]	1	Walking	2	Shank (bilateral)	ACC

^#^ Number, ACC accelerometer, BARO barometric pressure sensor, GYRO gyroscope, MAG magnetometer, RS reed switch

### Maintaining and changing a body position

#### Activities and outcome measures

The studies of this review often detected lying [26, 35, 37–50], sitting [26, 30, 31, 35, 37–59], and standing positions [26, 30, 31, 33, 35, 37–50, 52–56, 58–61] and, thus, estimated how long patients with mobility impairments maintain these positions in daily life. Some studies combined lying and sitting positions as sedentary behavior [33, 60–62]. One study included a measure to assess the knee angle during these positions [39]. Instead of quantifying the duration of body positions, it is also common to count the transitions between these positions. The transition between sitting and standing was frequently investigated [40, 41, 48, 53, 57, 59, 63–73], while only three studies detected the transition between lying and sitting [48, 57, 70]. Three of these studies further discriminated between transitions and bending forward [53, 65, 67], and two additional studies specifically detected sit-to-walk transitions since they aimed to compare the timed up and go test with transitions in daily life [74, 75]. Standing up was further analyzed in terms of speed [40, 63, 64, 68, 71, 73–75], range of motion [40, 64, 71, 74, 75], and smoothness [74, 75]. Only one study detected transfers (i.e., moving from one surface to another without changing body position) [62].

#### Description of algorithms and sensor placement

Activity classification algorithms in the literature detected either body positions directly or the transitions between them. Both approaches are widely used and, eventually, enable to determine how long a specific position was maintained and to count the number of transitions.

##### Detection of body positions based on sensor orientation

The orientation of different body parts are distinct characteristics of different body positions (e.g., the orientation of the thigh is vertical during standing, while it is horizontal during lying and sitting). Estimating the orientation of body-worn sensors and applying predefined thresholds is a common approach to discriminate between body positions in daily life. The sensors were placed on the thigh to distinguish between sitting and standing positions [31, 39, 40, 58, 60] as well as on the trunk [40] or shank [39, 58] to separate lying from the remaining positions. One study used the orientation of the pelvis to classify all three positions with a single sensor [46]. Algorithms to estimate the sensor’s orientation have already been summarized [76] and are, therefore, not part of this review.

##### Detection of transitions based on trunk inclination

Standing up or sitting down is usually performed by leaning forward to maintain the center of mass over the feet. This characteristic and the trunk inclination angle can be used to detect transitions between sitting and standing in daily life. The challenge is to discriminate between sit-to-stand and stand-to-sit transitions. This distinction was accomplished by pattern recognition [26, 41, 53, 64, 65, 67], by the orientation of the pelvis after the transition [74, 75], by the orientation change of the thigh during the transition [66, 68, 70, 72], and by estimating the difference in elevation with double integration of the acceleration signal in vertical direction [30, 35, 56, 63, 70, 73] or with a barometric pressure sensor [44, 69, 71]. Lying was often detected via the orientation of the trunk, as described above. Detecting lying and the transitions between sitting and standing requires only a single sensor on the trunk such as on the sternum [26, 26, 30, 35, 41, 44, 44, 56, 69–71], the waist [53, 65, 67], or the fifth lumbar vertebra [73–75]. Other studies used a trunk and a thigh sensor [33, 68, 70, 72] or just a thigh sensor [66], while the latter cannot discriminate between lying and sitting positions.

##### Measures to quantify body positions and transitions

The knee angle during lying, sitting, and standing was estimated with the differential signal of two sensors that were placed on the thigh and the ipsilateral shank [39]. No other measures were used in the literature to assess specific characteristics of different postures in daily life. Standing up, however, was more thoroughly analyzed. The start and end point of this transition were defined as the minima before and after peak trunk inclination. These points reveal the duration and with it a measure to quantify how fast patients are standing up. Five studies used a sensor on the sternum [40, 63, 64, 68, 71] and three a sensor on the fifth lumbar vertebra [73–75] to measure trunk inclination. Moreover, peak trunk inclination [64, 71], peak trunk acceleration [40], the range of acceleration [40, 64, 74, 75], and gyroscope signals [74, 75], as well as measures for smoothness [74, 75] were used to quantify standing up in daily life.

### Walking and moving

#### Activities and outcome measures

The studies included in this review most frequently covered detecting walking bouts in everyday life of people with mobility impairments [26–28, 30, 31, 33, 35, 37–58, 60, 61, 63, 68, 70, 72, 77–94], followed by more specifically detecting turning periods while walking [49, 68, 72, 79, 87, 88, 94, 95] and stair climbing [43–47, 50, 51, 54, 55, 59, 60, 83, 84, 87, 88, 96]. Other, less frequently detected activities were walking sideways [59], walking while carrying an object [87, 88], walking on sloping surfaces [47, 87, 88], and running [49, 51]. Several studies detected and counted steps during walking and stair climbing periods [26–28, 31, 33, 35, 39, 60, 63, 77, 79, 80, 86, 92, 93]. This in turn enables the estimation of step frequency and cadence. Walking bouts were further analyzed in terms of temporo-spatial gait parameters [27, 28, 33, 40, 58, 61, 70, 81, 82, 86, 91, 93], and joint kinematics (i.e. knee angle) [33, 39]. Turning periods were further analyzed in terms of duration [68, 72, 79, 87, 88, 94, 95], turning angle [79, 94, 95], turning speed [79, 94], smoothness [94], mediolateral range of trunk acceleration [94], and number of steps to complete a turn [79]. Stair climbing was often subclassified in ascending and descending [43–45, 54, 60, 83, 84, 87, 88, 96], and one study developed an algorithm that recognized if stairs were climbed with a step-by-step or a step-over-step pattern [83].

#### Description of algorithms and sensor placement

The following chapters describe the concepts of the underlying algorithm and the used sensor placement to detect and quantify walking, turning, and stair climbing activities. Details about the detection of walking sideways, walking while carrying an object, walking on sloping surfaces, and running as well as stair climbing with a step-by-step or step-over-step can be found in Table 5.

##### Walking bouts and gait parameters

Detection of walking bouts

Two approaches have been used in the studies included in this review to detect walking bouts of people with mobility impairments in unsupervised datasets. The first approach uses the signal magnitude or variance to discriminate walking from static activities such as sitting and standing. The data is labeled as walking if the signal exceeds a predefined threshold for a certain duration. For this purpose, studies used the acceleration signal of the pelvis [27, 81, 82, 86], thigh [80], shank [68, 72], thigh and shank [39], or the angular rate of the pelvis [79, 94]. Some studies introduced additional criteria to avoid confusion with other activities. During valid walking bouts, the orientation of the pelvis [27, 86] or thigh sensor [80] needs to be vertical or the hip angle, derived from the differential signal between the pelvis and the thigh sensors, needs to be in an extended position [68, 72]. The second approach more specifically detects steps in the signal, and a number of consecutive steps are seen as a walking bout. The initial contact of each step leads to a peak in the signals and these peaks appear with a certain frequency that is specific to walking. Thus, peak detection and optionally verifying if they appear within a predefined frequency band is a common method to detect steps in unlabeled data. This method has been implemented with the acceleration signal of the trunk [28, 30, 35, 44, 56, 63, 70, 78, 92], pelvis [27, 86, 92], thigh [31, 60, 80], ankle [91] or foot sensor [40], as well as the gyroscope signal of the shank [26, 33, 41, 70] or foot sensor [79]. Again, to reduce false-positive rates, peak detection has been combined with the vertical orientation of the trunk and thigh sensors while walking [40]. Another method to detect steps is to assess the similarity of the signal to pre-established templates. The similarity was assessed with dynamic time warping of the feet’s gyroscope signal [77] and with cross-correlation of the shank’s acceleration signal [39]. A third method used the fact that the left and right foot are alternatively active and stationary during walking. Active and stationary phases were detected with a zero-velocity algorithm and by fusing the accelerometer and gyroscope signal of the feet sensors [58]. Some studies used the first approach to detect walking bouts and the second to detect steps within these walking bouts, while two studies combined both approaches to detect walking bouts more specifically [53, 93]. The detection of walking bouts enables to measure the number and duration of walking activities in everyday life, while the detection of steps, further, enables to count daily steps as well as to determine the cadence [26, 28, 31, 78, 92], stride time [91], and stride time variability [28, 78] of individual walking bouts. Besides, the cadence was also determined by frequency analysis of the acceleration signal without detecting each step individually [92, 93].

Determination of gait parameters

Deriving temporal gait parameters from previously detected walking bouts, such as the duration of stance, swing, and double support phase requires a segmentation of the gait cycle by identifying the initial and final contact of the feet with the ground. Three different approaches were used in the literature to identify these gait events in people with mobility impairments. The first approach assumes that the lower leg rotates forwards during the stance phase and backwards during the swing phase. Zero-crossings of the feet’s gyroscope signal around the mediolateral axis before and after maximal backward angular rate (i.e., swing phase) were, therefore, detected to estimate the timing of the final and initial contacts, respectively [79]. As an alternative to zero-crossings, the maxima of forward angular rate were detected to estimate the timing of the gait events. This algorithm was applied to the gyroscope signal of the feet [40] or the ankle sensors [33, 70]. The second approach used distinct features of the pelvis’ acceleration signal in a vertical direction. It was assumed that the initial contact corresponds to peak deceleration, while the final contact does to peak acceleration gain [27, 86]. The third approach determines the start and end points of the stationary phase (i.e., stance phase) of the feet sensors [58]. Again, the stationary phase was detected with a zero-velocity algorithm.

Walking speed was derived directly by estimating the stride length and divide it by the stride time or indirectly by identifying a surrogate that correlates with walking speed. The stride length was determined with biomechanical models and kinematic chains to estimate the distance between the two feet, or with the inverted pendulum model in which the stride length can be derived from the height change of the center of mass, or with double integration of the feet’s horizontal acceleration [40]. The biomechanical models required IMUs on both thighs and shanks [33, 70] as well as additionally on the pelvis and the feet [58], while the inverted pendulum model only needs the vertical acceleration signal of the pelvis [27, 86]. Several surrogates that are supposed to correlate with walking speed were described in the studies of this review. Namely, the root mean square of the acceleration signal at the pelvis [93], or of the vertical velocity of the trunk [28, 78] as well as the stride time [91]. Moreover, one study recognized comfortable and brisk walking as two distinct classes, which enables a dichotomous analysis of slow and fast walking speed [61].

Walking bouts were further analyzed regarding stability, foot clearance, and joint kinematics. Gait stability as a measure for risk of falling was determined with local dynamic stability [81, 93] and entropy measures [82] of the pelvis’ acceleration signal. The knee angle was measured with the differential signal between the thigh and ankle sensors [33, 39]. And one study estimated the foot clearance with the position of the foot sensor [58].

##### Turning

Turns during walking bouts were detected whenever the turning angle or angular velocity around the vertical axis exceeded a predetermined threshold. The turning angle was derived from the trunk [68, 72] or the pelvis sensor [49, 79, 94, 95]. The detection of turns enables to count the number of turns in daily life. However, to derive other measures, the start and end point of these turns need to be detected, too. These time points were defined when the angular velocity of the pelvis sank below a predetermined threshold [79, 94], or at the minima before and after peak turning angular velocity of the trunk [68, 72], or at the minimum and maximum of the pelvis’ turning angle [95]. Knowing the start and end point of turning periods enables to determine its duration [68, 72, 79, 94, 95], turning angle [79, 94, 95], and turning speed [79, 94] as well as the smoothness [94], mediolateral range of trunk acceleration [94], and the number of steps to complete a turn [79].

##### Stair climbing

The range of motion at the hip joint is higher during stair climbing compared to level walking. This characteristic was used in two studies to recognize stair climbing activities in daily life. One study used the orientation of the thigh sensor to discriminate between stair climbing and level walking [60], while another one used the variance of the acceleration signal at the hip [47]. A further distinct characteristic of stair climbing is the change in altitude. Several studies used a barometric pressure sensor to measure the altitude change during locomotion and discriminated between going up and down stairs as well as level walking [44, 45, 84]. Usually, the shank is rotating forward during the stance phase of walking trials. However, while ascending a flight of stairs, there is a period during the stance phase, in which the shank is rotating backward. One study used this fact to specifically recognize stair ascending periods with the gyroscope signal of the shank sensor [96]. And lastly, one article used the timing of peak occurrence in the acceleration signal of the thigh sensor to discriminate between ascending and descending stairs [60].

### Moving around using a wheelchair

#### Activities and outcome measures

The included articles in this review either specifically detected active self-propulsion of wheeling activities [97, 98] or discriminated between active self-propulsion and being pushed passively [17, 29, 32, 62, 99–101]. Studies that did not distinguish between active and passive wheeling bouts were not included in this review since they did not specifically address a motor activity. Active wheeling was further analyzed in terms of covered distance [29, 32], speed [17, 29] as well as the number of strokes and stroke frequency [102]. Moreover, three studies allocated wheeling bouts either to maneuvering or covering longer distances [17, 29, 32], five studies differentiated between hand use during self-propulsion and other activities of daily living [29, 62, 99–101], and one study detected playing basketball [101].

#### Description of algorithms and sensor placement

Many studies used a statistical machine learning approach and are already depicted in Table 5. The remaining concepts of the underlying algorithms and used sensor placements are described in the following section.

Wheeling bouts were detected by measuring the rotation of the wheel and setting predefined thresholds. The rotation of the wheel was measured with a gyroscope [29, 32] or a reed switch [102] on the spokes of the wheelchair. The distinction between maneuvering and longer wheeling bouts was accomplished with two different approaches. The first approach simply defined wheeling bouts that are shorter than 5.12 s as maneuvering and the remaining bouts as longer wheeling bouts [32]. The second approach used the acceleration signal of the wheel sensor and predefined, incremental thresholds to distinguish between non-wheeling bouts, maneuvering, as well as normal speed and high-speed bouts [17]. Two studies separated active from passive wheeling propulsion whenever the acceleration signal of the wrist sensor exceeded a predefined threshold [17, 97]. Another study specifically counted the number of strokes within wheeling activities and, with it, estimated the stroke frequency by means of peak detection of the acceleration signal of the upper arm, wrist, or wheelchair sensor [102]. Besides, the speed and distance of active wheeling bouts were estimated by measuring the angular velocity and the radius of the wheel [29, 32].

### Upper extremities

#### Activities and outcome measures

The measures to quantify hand and arm use in daily life that were used in the studies of this review were allocated to one of the following three categories: (1) Non-specific hand and arm use regardless of the underlying activity, (2) specific hand and arm movements such as reaching, and (3) specific hand and arm activities that require a combination of movements (e.g., eating activity involves reaching, cutting, and lifting movements). The first category includes measures to quantify the amount [30, 40, 46, 47, 89, 103, 104] and diversity [105] of hand and arm use as well as the range of motion of shoulder [34, 56, 58, 106], elbow [34, 58], and hand movements [107]. The second category contains reaching [34, 58, 72, 108–111], lifting [57, 108–111], and pouring (i.e. pro- and supination) movements [108–111], while reaching was further analyzed in terms of reaching distance [34, 58] and reaching direction [72]. And the activities of the last category were writing and reading [42], opening a door [87, 88], hair combing [57, 112], eating [42, 112, 113], and drinking [112] as well as tooth brushing, shirt buttoning, pant lifting, and food cutting [57].

#### Description of algorithms and sensor placement

##### Non-specific hand and arm use

Hand and arm use in daily life is often measured with activity counts that are derived from the accelerometer signal of the wrist sensors. Applying a sensor on either side enables to estimate the hand use laterality, which is particularly relevant for people with unilateral impairments. Studies that based their outcomes solely on activity counts were not included in this review since they do not provide innovation to the state-of-the-art and are already well investigated and reviewed in the literature [114, 115]. Instead of measuring the amount of hand and arm use, one study included in this review developed an algorithm do determine the diversity of hand and arm movements by calculating the sample entropy of the upper and lower arm acceleration signals [105]. Still, the signals of sensors worn at the upper extremities are biased by movements of the lower extremity (e.g., walking leads to large numbers of activity counts at the wrists even though the arms are not actively used) and three approaches are described in the literature to overcome this issue. The first approach stratifies hand and arm use according to the underlying activity of the lower extremities (e.g., hand and arm use during sitting, standing, and walking). This enables the exclusion of passive arm swing while walking [30, 40, 46, 47]. The second approach directly discriminates between functional and non-functional hand and arm use. This distinction was implemented by training a classifier with machine learning techniques (see Table 5 for details about sensor type and placement) [103, 104] and by limiting the range of functional hand movement [89]. Here, functional hand movement was defined whenever the orientation of the hand was within ± 30° from the horizontal, and the range of hand movement in this section exceeded 30° in a 2 s period. The orientation of the hand was determined with an IMU on the wrist. And lastly, the third approach estimated the movement of specific joints of the upper extremities. Shoulder movement was determined by calculating the angle between the trunk and the upper arm sensor [34, 58], by estimating the arm elevation with the orientation of the upper arm sensor [56], and by assessing the spatial distribution of the elbow position with a kinematic model and the orientation of the upper arm sensor [106]. Likewise, the elbow movement was determined by calculating the angle between the upper and lower arm sensors [34, 58], while the wrist and finger movements were detected with an IMU (incl. magnetometer) on the wrist and a magnet on the index finger [107].

##### Specific hand and arm movements

A more sophisticated approach to discriminate between functional and non-functional hand and arm use is to detect particular movement primitives such as reaching an object. One research group developed an algorithm that distinguishes between reaching, lifting, and pouring movements while making a cup of tea by using a single wrist sensor [108–111]. Another study specifically detected lifting food towards the mouth [57], and three studies detected reaching movements [34, 58, 72]. These studies used a whole-body IMU system with up to 17 sensors, which raises questions about its applicability for long-term measurements in daily life. Reaching movements were further analyzed by measuring its range and direction with the difference between the hand and trunk positions [34, 58] and by classifying the movement into upwards, mid, and downwards reaching directions [72].

##### Specific hand and arm activities

All but one study and most of the activities of this category were detected with a statistical machine learning approach. The details about sensor placement are presented in Table 5. One study used a pattern recognition approach with template matching to discriminate between hair combing, eating, and drinking [112]. The templates were based on the signals of seven IMUs (incl. magnetometer), and they were placed on the trunk as well as on the upper arm, forearm, and hand of each side.

## Discussion

This systematic review focused on the application of inertial sensor technologies to quantify everyday life motor activity in people with mobility impairments and provides an overview of existing outcome measures. It, further, describes the concepts of the underlying data processing algorithms as well as the types and placements of required sensors to derive these measures and, eventually, lists the designs and populations of all studies that evaluated the measures in terms of validity, reproducibility, and usability.

The included studies of this review covered a large variety of outcome measures and underlying data processing algorithms which can be grouped into four categories: (1) maintaining and changing a body position, (2) walking and moving, (3) moving around using a wheelchair, and (4) activities that involve the upper extremity. The validity or reproducibility of these outcomes measures was investigated in fourteen different patient populations, of which the majority comprised stroke survivors, patients with Parkinson’s disease, and patients with spinal cord injury. Most of the studies evaluated the algorithm’s accuracy to detect certain activities in unlabeled raw data, while others evaluated the outcome measures in terms of concurrent validity, discriminant validity, or reproducibility or applied them in an interventional or observational study. The type and placement of required sensor technologies depends on the activity and outcome measure and are thoroughly described in this review. The reproducibility of the outcome measures and the usability of the applied sensor setups were rarely reported.

This review is limited to applications of wearable inertial sensors that were optionally combined with other sensor technology. However, among the included articles, there were two measurement tools that have the potential to monitor everyday life motor activities without combining it with inertial sensors: insoles with force-sensitive sensors [18–23] and first-person cameras [24, 25]. Even though instrumented insoles are reliable gait phase detectors [20], their applicability for long-term measurements in daily life is limited since the user might change or take off the footwear during the measurement period, which in turn would lead to biased outcome measures. First-person cameras might be superior to inertial sensors from a technological perspective since they also provide information about the user’s environment and social interactions [116]. However, the application of wearable cameras in daily life also raises ethical questions, and it remains to be seen whether this technology will be accepted by the end-users and the community. Other technologies, such as external cameras, pressure-sensitive walkways, or instrumented furniture, could be used to quantify motor activities in daily life. Even though these technologies would allow for an in-depth analysis of motor activities, they are all limited to a specific area and, therefore, not feasible to monitor the patients' activities throughout the day. Consequently, we are still convinced that wearable inertial sensors are the preferred measurement tool to monitor everyday life motor activities in patients with mobility impairments. Amongst wearable sensors, accelerometers were the preferred technology in the articles of this review. Compared to gyroscopes, accelerometers do have a considerably lower power consumption [117] and are not susceptible to drift [12], which might explain their preference for unobtrusive long-term measurements in daily life.

The search strategy and eligibility criteria of this review were designed to get an overview of all reproducibly described algorithms that process unlabeled raw data of everyday life measurements into clinically meaningful outcome measures. Despite this systematic search, there are three reasons why the algorithms and outcome measures of this review are incomplete. First, proprietary algorithms of commercial devices and insufficiently described algorithms were not considered in this review, even though they might determine clinically meaningful outcome measures. Transparency of scientific methods (including the data processing algorithm) enables other researchers to interpret the results, to validate the method, and to replicate the study, which is essential to the development and evolution of science [118]. We, therefore, encourage the scientific community to use open-source algorithms or at least describe the used algorithm reproducibly. Second, only algorithms that are applicable to unlabeled raw data were included in this review, and, especially in the field of gait analysis, there are many algorithms available that determine a clinically meaningful outcome measure out of labeled walking trials [119]. These algorithms could be combined with an activity/walking detection algorithm and, thus, extend the variety of outcome measures to quantify everyday life motor activities. And third, algorithms that were evaluated in healthy subjects were not considered in this review, but might as well provide clinically meaningful outcome measures. However, whether these algorithms also work correctly in patients with mobility impairments, has to be shown in future research.

Neither a quality assessment of the included studies nor a meta-analysis regarding the accuracy or reproducibility of the described algorithms and outcome measures were conducted in this review. Although we acknowledge the benefit of these analyses, they are not feasible for the current review due to missing standards to assess the quality of activity classification studies and due to the large heterogeneity of the methods and data reporting of the studies. For example, we included two studies that evaluated an algorithm to detect walking and stair climbing in stroke survivors [46, 84]. Even though these studies had a similar study population and study design, their algorithms' performance is still not comparable since their algorithms detected three and six activities, respectively, and the authors chose different metrics to report their results. One study reported sensitivity and specificity, while the other study reported F-scores. This example demonstrates the difficulty of determining which algorithms are superior, and the comparability between studies is even more complicated when the study population and study designs differ. We, therefore, encourage the scientific community to develop a standard to conduct such studies and to report the results consistently. We suggest that the study protocol either contains observations of the patients' daily motor activities in their habitual environment or an activity circuit that resembles everyday life and comprises activities not classified by the algorithm. We further recommend that the confusion matrix is reported, which allows determining a large variety of statistical measures to quantify the algorithm's performance. Moreover, we would like to point out the difference between measurement error and activity classification accuracy. Detecting sitting position with an accuracy of 90%, for example, does not necessarily mean that the error of estimating the sitting duration of a 24-h measurement is 10%. In fact, a balanced occurrence of false positive and false negative detections would lead to a much smaller error. Although the measurement error is essential for future applications of the algorithm to daily life data, it is rarely reported in the literature. Therefore, we recommend future studies to determine the measurement error of their outcome measures instead of just reporting the activity classification accuracy.

The usability of wearable inertial sensors was hardly ever assessed or at least not reported in the studies of this review article. This finding is somewhat surprising since the end user’s compliance and acceptance to wear the sensors throughout the measurement period is crucial to get comprehensive and unbiased data of the end user’s motor activities in daily life. We believe that the usability of the sensor system depends predominantly on the number and size of sensors, on the location of sensor placement, and on how the sensors are attached to the body. Moreover, low usability of the sensor system might also interfere with the end-user’s behavior in daily life. However, this has yet to be shown, and we, therefore, recommend that future studies consequently report the wearing time and the obtrusiveness of their sensor system.

## Conclusions

This systematic review provides a comprehensive overview of applications of wearable inertial sensors to quantify everyday life motor activity in people with mobility impairments. It lists activities and outcome measures that have been covered in the literature and describes the concepts of the underlying data processing algorithms as well as the required sensor technologies. It, further, tabulates the study populations and the study designs of the included articles. This review, therefore, summarizes the state-of-the-art of existing sensor applications, it provides quick access to the relevant literature to the reader that is interested in quantifying certain activities in a specific patient population, and it enables the identification of gaps for the evaluation of existing and the development of new algorithms.

The studies of this review had a large methodological heterogeneity and reported their results inconsistently. This made it impossible to quantify and compare the validity, reproducibility, and usability of different sensor technologies, its underlying algorithms, and their outcome measures. Thus, this review neither provides recommendations about the favored type and placement of sensor technologies, nor a synthesis about the performance of different algorithms. Therefore, we recommend that future studies follow a standardized protocol and use consistent metrics to report their results.

In the literature, wearable inertial sensors are the preferred technology to monitor everyday life motor activities in patients with mobility impairments. We further expect the use of this technology to evolve substantially as more and more valid algorithms become available for patient populations that can capture different facets of everyday life, as can be seen in the healthy population.

## Data Availability

Data sharing is not applicable to this article as no datasets were generated or analyzed during the current study.

## Abbreviation

IMU: Inertial measurement unit
